# Supplementary material for: A UMLS-based spell checker for natural language processing in vaccine safety
Source: BMC Med Inform Decis Mak. 2007 Feb 12;7:3. doi: 10.1186/1472-6947-7-3 (PMC1805499; doi:10.1186/1472-6947-7-3)
Supplement: Additional file 13 — RAP application directory. Sets up directory for RAP (RDF application for PHP) source code [file 1472-6947-7-3-S13.gz › rap/doc/tests/test_SerializerOptions.php.txt]

Test Serializer Options

php
define("RDFAPI\_INCLUDE\_DIR", "./../api/");
include(RDFAPI\_INCLUDE\_DIR . "RDFAPI.php");
include(RDFAPI\_INCLUDE\_DIR . PACKAGE\_SYNTAX\_RDF)
// Filename of an RDf document
$base="example1.rdf";
// Create a new MemModel
$model = ModelFactory::getDefaultModel();
// Load and parse document
$model-load($base);
// Output model as HTML table
$model->writeAsHtmlTable();
echo "

";
// Create Serializer and serialize model to RDF with default configuration
include(RDFAPI\_INCLUDE\_DIR . PACKAGE\_SYNTAX\_RDF);
$ser = new RDFSerializer();
$rdf =& $ser->serialize($model);
echo "

" . $rdf . "";
// Serialize model to RDF using attributes
$ser->configUseAttributes(TRUE);
$rdf =& $ser->serialize($model);
echo "

" . $rdf . "";
$ser->configUseAttributes(FALSE);
// Serialize model to RDF using entities
$ser->configUseEntities(TRUE);
$rdf =& $ser->serialize($model);
echo "

" . $rdf . "";
$ser->configUseEntities(FALSE);
?>
